# Preliminary Structural Characteristics of Polysaccharides from Pomelo Peels and Their Antitumor Mechanism on S180 Tumor-Bearing Mice

**DOI:** 10.3390/polym10040419

**Published:** 2018-04-09

**Authors:** Juan Yu, Haiyu Ji, Anjun Liu

**Affiliations:** Key Laboratory of Food Nutrition and Safety, Ministry of Education, College of Food Engineering and Biotechnology, Tianjin University of Science and Technology, Tianjin 300457, China; yujuan14615@163.com (J.Y.); haiyu11456@163.com (H.J.)

**Keywords:** pomelo peels polysaccharides, structural characteristics, antitumor activity, immunoregulatory function

## Abstract

In this study, the polysaccharides (PPs) from pomelo peels were investigated for their structural characteristics and antitumor mechanism on sarcoma S180-bearing mice. Components, FT-IR, and GC analysis showed that PPs, mainly composed of glucose, were typical acid polysaccharides with α-d-pyranoid glucose containing 74.52% carbohydrate and 16.33% uronic acid. The in vivo antitumor tests revealed that PPs could effectively suppress the transplanted S180 tumors growth, as well as protect the immune organs, improve proliferation ability of splenic lymphocytes and killing activity of NK cells in tumor-bearing mice. Furthermore, the levels of serum cytokines (IL-2, IFN-γ and TNF-α) and the proportion of CD4^+^ T cells in peripheral blood of mice bearing S180 tumors were also significantly increased after treatment with PPs. Meanwhile, the transplanted S180 tumor cells exhibited obvious apoptotic phenotype after PPs treatment by arresting the cell cycle in S phase, down-regulating the Bcl-2 expressions and up-regulating the Bax levels. These data showed that PPs were mainly composed of glucose with α-d-pyranoid ring and could induce apoptosis of solid tumor cells by enhancing the antitumor immunity of tumor-bearing mice, which would provide a theoretical basis for the practical application in food and medical industries.

## 1. Introduction

Cancer has become the major threatening factors for human beings with a high incidence and mortality [1]. The World Health Organization (WHO) made an official announcement that the percentages of patients died from cancer in 2012 have risen by 40% in the past decade. Chemotherapy has been confirmed to have cytotoxic effects on tumor cells, however, these chemotherapeutic drugs could also impair the patients’ normal cells and lead to cancer cells’ therapeutic resistance, which can cause lots of unexpected side effects and influence the survival probability [2,3]. Therefore, it is extremely urgent to search for a novel natural anticancer drug with higher bioactivities and lower toxicity.

Accumulating evidence has demonstrated that immune system plays a vital role in immunosurveillance against malignant cells, and an effective induction of tumor-specific immune response can greatly strengthen the antitumor activity of a drug [4,5]. Thus, boosting the immunity of an individual is helpful for the therapy of malignant tumors. Polysaccharides, a kind of biological response modifier (BRM), have become one of the research focuses in the past decades because of their broad spectrum of biological effects, such as antioxidant activity, differentiation-inducing activity, as well as antitumor activities via activation of the relevant immune responses of the host [6,7]. Numerous studies have shown that polysaccharides have the potential to exert anti-cancer effects by several underlying mechanisms, such as the direct toxic effect on the cancer cells via inducing cellular apoptosis and cell cycle arrest, or immuno-enhancing effect on the host organism by oral administration of active ingredients, thereby preventing tumor formation, invasion, and metastasis [8,9]. Furthermore, the polysaccharides might be synthesized and modified for higher purity and stronger antitumor activity, which would help to clarify drug delivery systems [10]. In view of this, tremendous efforts should be made to discover the bioactive polysaccharides from natural materials for the development of potential prophylactic and therapeutic agents of various cancers.

Pomelo (*Citrus grandis Osbeck*), one of the most important *citrus* fruit in Rutaceae family native to China and parts of Southeast Asia, has won masses of consumers at home and abroad because of its unique flavor and potential nutritive value [11]. The pomelo is widely cultivated and their annual production exceeded millions of tons in China. The pomelo peels which account for approximately 30% of gross weight of the fruit possess high medicinal value and health function, which are recorded in the 2010 version of the “Chinese Pharmacopoeia” [12]. At present, the researches about pomelo peels are mainly focused on the extractive techniques of essential oil, pectin, naringin and phenolic compounds [13,14,15,16], as well as their relevant antibacterial, antidiabetic, antitumor, antioxidant and lowering serum cholesterol effects [17,18]. As we all know, polysaccharides, one of the prominent active components in pomelo peels, may have stronger biological activities without any damage to the host due to their good water solubility and leachability compared with saponins and essential oil [19]. For this reason, it is beneficial to make full use of the polysaccharides from pomelo peels for the maintenance of health and lower risk of chronic disease including cancer. Although many acidic polysaccharides derived from plants have been reported to have the remarkable antitumor immune responses [20,21], the comprehensive researches of bioactive polysaccharides from pomelo peels on the structural characterization and in vivo antitumor activity remain still unknown. Accordingly, there have been great interests in exploring the promising application of pomelo peels polysaccharides.

The aim of present study was to extract the polysaccharides (PPs) from pomelo peels and evaluate their antitumor and immunomodulatory activities against transplanted S180 sarcoma cells, and attempted to investigate the underlying mechanism in depth. These results will be valuable to provide the scientific basis for the development of functional food and potential alternative anticancer medicine.

## 2. Materials and Methods

### 2.1. Plant Materials

The pomelos (*Citrus grandis* (L.) Osbeck) were harvested from Pinghe country of Zhangzhou city (Fujian, China) in November 2014, and first washed with deionized water thoroughly to remove all dirt and other contaminants. The pomelo peels were collected by manual shaving, dried to constant weight in an oven at 50 °C, screened through 80 mesh sieve to obtain the homogeneous powder sample, and finally stored under the suitable conditions for subsequent study.

### 2.2. Standards and Reagents

S180 cells (murine sarcoma cells) were bought from the Shanghai Institute of Biological Sciences at the Chinese Academy of Sciences (Shanghai, China). Bovine serum albumin (BSA), standard monosaccharides, glucuronic acid, trifluoroacetic acid (TFA), 3-(4,5-dimethylthiazol-2-yl)-2,5-diphenyltetrazolium bromide (MTT), dimethyl sulfoxide (DMSO), and 5-fluorouracil (5-FU) were obtained from Solarbio (Shanghai, China). Pectinex BE XXL (16,000 PGU/mL) were obtained from Novozymes biotechnology Co., Ltd. (Tianjin, China). Medium RPMI-1640 was provided by Hyclone (Thermo Scientific Inc., Waltham, MA, USA). Fetal bovine serum (FBS) was purchased from Hangzhou Sijiqing Corp (Hangzhou, China). Cytokine detecting ELISA kits were bought from Lufeng Bio-Technique Co., Ltd. (Shanghai, China). PIPA, Bradford Protein Assay Kit, Enhanced chemiluminescence (ECL) kit were obtained from Solarbio Science &Technology Co., Ltd. (Beijing, China). The antibodies specific to β-actin, Bax and Bcl-2 were provided by Tianjin Sungene Biotech Co. (Tianjin, China). All other chemicals and agents were of analytical grades.

### 2.3. Extraction Procedure of PPs

The pretreated powder (100 g) of pomelo peels was extracted three times with distilled water (3 L) for 2 h at 90 °C. After extraction, the water extracts were filtered and centrifuged to remove the insoluble fractions. The obtained supernatant was concentrated and precipitated by adding 4 volumes of ethanol. The precipitations were obtained after centrifugation at 4500 rpm for 15 min, and then washed by anhydrous ethanol and acetone alternately 3 times to remove the lipids and pigments thoroughly. Afterwards, the crude samples (~7.33 g per 100 g dry weight) were re-dissolved in deionized water, the pectin was eliminated by addition of 0.2% pectinase (pH 4.5) at 55 °C for 70 min, and heated at 90 °C for 10 min. After treatment, the mixture was neutralized with 0.1 M NaOH, followed by concentration and deproteination according to Sevag method with 1-butanol and chloroform (1:4, *v*/*v*) for 5 times. Finally, the mixture was dialyzed (MWCO 3500) against distilled water for 72 h to exclude the salt and small peptide, purified via freeze thawing method to further remove proteins and other unstable substance [22,23], and lyophilized to get the pomelo peels polysaccharides (PPs, ~4.45 g per 100 g dry weight) for further study. 

The extraction yield (Y) of PPs was calculated as follows:

Y (%) = weight of PPs (g)/weight of pretreated pomelo peels powder (g) × 100.

### 2.4. Physicochemical Properties Analysis

The total sugar content was measured by the phenol-sulfuric method using glucose as the standard. The protein content was quantified by the Coomassie brilliant blue method using bovine serum albumin as the standard [24]. The content of uronic acid was detected according to the carbazole-sulfuric method using glucuronic acid as the standard [25].

The samples (5 mg) were dissolved and diluted into 1 mg/mL, and scanned from 200 to 800 nm with a spectrum-2102 UV spectrophotometer. FT-IR spectrum was performed with a Fourier transformed infrared spectrophotometer (Bruker VECTOR-22, Karlsruhe, Germany) in the wavelength range of 4000–400 cm^−1^ using the method of KBr pellet. The detailed procedure was as followed: 0.7 mg of sample was mixed with 150 mg of dry KBr and pressed into a disk for the infrared spectroscopic analysis.

The monosaccharide composition was determined and quantified by gas chromatography (GC). The polysaccharides samples (5 mg) were hydrolyzed in 1 mL of 2 M trifluoroacetic acid (TFA) at 110 °C for 4 h in sealed tubes. After the removal of TFA, the hydrolysate was acetylated and analyzed by GC (GC2010, Shimadzu, Kyoto, Japan) [26]. d-arabinose, d-glucose, d-galactose, d-xylose, d-mannose and l-rhamnose were derivatives used as the standards. 

### 2.5. Antitumor Activity In Vivo

#### 2.5.1. Design of Animal Model

Female Kunming mice (6–8 weeks old), weighing 18–22 g, were purchased from the Center of Experimental Animals of Academy of Military Science (Beijing, China). All animals were raised under pathogen-free conditions with a controlled temperature (20–25 °C) and a relative humidity (50 ± 5%). All mice were allowed to acclimate to new surroundings for a week prior to experiments. They were allowed free access to tap water and fed with standard pellet diet on a 12-h light/dark cycle throughout the experimental period. All animal experimental procedures were conducted in accordance with the principles of Laboratory Animal Care and approved by the Local Ethics Committee for Animal Care and Use at Tianjin University of Science and Technology.

The healthy mice were randomly divided into five groups of 10 mice each group: blank group, model group, PPs groups (100 and 300 mg/kg PPs) and positive group (5-FU, 20 mg/kg). The mice in PPs and positive control groups were orally administrated with two doses of PPs (100 and 300 mg/kg body weight) or 5-FU (20 mg/kg body weight) once a day. For the blank and model groups, the mice were given the same volume of normal saline by intragastric administration. After 15 days of gavage, all mice except the blank group were injected subcutaneously in armpit of right hind limb with S180 tumor cells (2 × 10^6^ cells/mouse) and treated intragastrically for another three weeks. The tumor size was observed and measured by using vernier caliper every two days throughout the experimental period.

All the mice were weighed and then sacrificed by cervical dislocation after the last dose. The tumors, spleens and thymuses were collected carefully and weighed immediately for further studies. The tumor inhibitory ratio was expressed by the following formula: Inhibitory rate (%) = (the average tumor weight of model group—he average tumor weight of treated group)/the average tumor weight of model group × 100. The immune organs indexes were expressed as the organ weight relative to body weight [7]. 

#### 2.5.2. Splenic Lymphocyte Proliferation Assay

Splenic lymphocyte proliferative activity was performed by MTT method as described previously with little modifications [27]. Briefly, the prepared splenocytes (1 × 10^6^ cells/well) in 96-well plates were exposed to Con A (final concentration is 5 μg/mL), LPS (final concentration is 10 μg/mL) or RMPI 1640 complete medium. After incubation for 48 h, the number of cells was determined by MTT assay using a microplate reader (Model 680, Bio-Rad, Hercules, CA, USA), and the stimulation index (SI) was determined by the following formula:

Stimulation index (SI) = OD_1_/OD_0_, where OD_0_ is the OD value of splenic lymphocytes exposed to RMPI 1640 complete medium, OD_1_ is the OD value of splenic lymphocytes exposed to Con A or LPS.

#### 2.5.3. NK Cells Activity Analysis

The freshly prepared splenic NK cells obtained by NK cells isolation kit II (Miltenyi Biotec, Bergisch Gladbach, Germany) were used as effector cells and S180 cells were used as target cells. NK cells and S180 cells at the ratio of 20:1 were co-cultured with RPMI 1640 medium in 96-well plates and they were also incubated separately with the medium as controls. After incubation for 48 h, the optical density (OD) was determined by MTT assay. The cytotoxic activity of NK cells was calculated with the following equation:

NK cytotoxic activity (%) = (OD_0_ + OD_1_ − OD_2_)/OD_0_ × 100%, where OD_0_ is the OD value of target cells, OD_1_ is the OD value of effector cells, OD_2_ is the OD value of the co-cultured cells.

#### 2.5.4. Evaluation of Serum Cytokines in Mice Serums

The serum was taken from mice, collected immediately by centrifugation at 4000 rpm for 15 min at room temperature and then stored at −80 °C [28]. IL-2, IFN-γ and TNF-α ELISA kits were employed to evaluate their cytokines levels according to the manufacture’s protocol.

#### 2.5.5. Assessment of Lymphocyte Subsets in Peripheral Blood

FCM assay was used to characterize the phenotype of lymphocytes in peripheral blood. The cells were obtained and stained with monoclonal antibodies (mAb) against CD3^+^, CD4^+^ and CD8^+^ for 30 min on ice in the dark. The fluorescently-labeled lymphocytes were re-suspended and the antigen expression was measured using flow cytometry (BD FACSCallibur, Becton Dickinson, Franklin Lakes, NJ, USA). The results were analyzed by CellQuest Pro software (version 5.1, Becton Dickinson, Franklin Lakes, NJ, USA) and expressed as the percentage of positive cells within the total detected lymphocytes.

### 2.6. Apoptosis Detection of Tumor Tissues

#### 2.6.1. Histological Observations of Tumor Tissues

The tumor tissues of the mice were fixed in 10% neutral formaldehyde solution and dehydrated in gradient ethanol solution. After embedding in paraffin, 4-μm sections were obtained and stained with hematoxylin and eosin (H&E) for microscopic examination.

#### 2.6.2. Cell Cycle Analysis Using Flow Cytometer

The tumor cells suspension were prepared and fixed with cold 70% ethanol for at least 18 h at 4 °C. Subsequently, the fixed cells were subjected to PI/RNase Staining Buffer for 20 min. The stained cells were analyzed by flow cytometer (FACS Cablibur, Becton Dickinson, Franklin Lakes, NJ, USA).

#### 2.6.3. Western Blotting

The tumor tissues were lysed with RIPA buffer according to the manufactures’ directions. The concentrations of protein were quantified using BCA assay kit. Bax, bcl-2, and β-actin protein expression were detected by western blotting with the rabbit monoclonal antibody to Bax (1:1000), Bcl-2 (1:1000) and mouse monoclonal antibody to β-actin (1:10,000) as the primary antibodies. The blots were determined using enhanced chemiluminescence (ECL, West Bengal, India) assay and exposed to X-ray imaging films in a darkroom. The band density were normalized to internal control β-actin and analyzed with Quantity One software (version 4.6.2, Bio-Rad Laboratories, Inc., Hercules, CA, USA).

### 2.7. Statistical Analysis

All values were presented as the mean ± standard deviation (S.D.). Statistical analyses of these data were performed using SPSS for windows, version 19.0 (SPSS Inc., Chicago, IL, USA). The significance of difference was analyzed by one-way analysis of variance (ANOVA) followed by the Duncan’s test. The data was considered to be significant at *p* < 0.05.

## 3. Results

### 3.1. Physicochemical Characteristics of PPs

**Figure 1:**
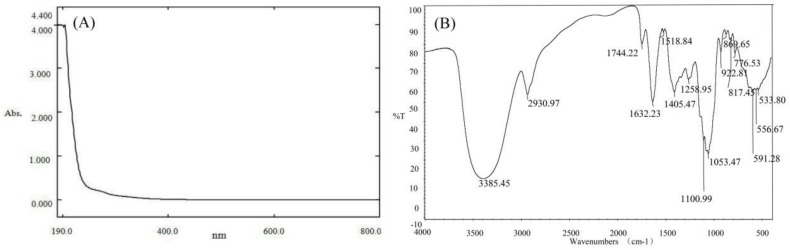
UV spectra of PPs in the range of 190–800 nm with a spectrum-2102 UV spectrophotometer (**A**) and FT-IR spectra of PPs in the wavenumber range of 4000–400 cm^−1^ with a Fourier transformed infrared spectrophotometer using KBr pellet method (**B**).

The PPs was obtained from pomelo peels by the conventional hot water (90 °C) extraction and 80% alcohol precipitation. After the removal of pectin and protein, dialysis and lyophilization, the extraction yield of polysaccharides was calculated as 4.45%, and our previous study proved that the average molecular weight of PPs was 1.1 × 10^5^ Da. The content of total sugar, uronic acid and protein in PPs was 74.52%, 16.33% and 3.56%, respectively, based on the chemical analysis. UV–vis absorption spectra of PPs showed no absorption at 280 and 260 nm, indicating PPs contain no or trace amount of nucleic acid and protein in PPs (Figure 1A), consistent with the determination of protein content (3.56%).

As shown in Figure 1B, the IR spectrum was conducted to investigate the functional groups of PPs. The polysaccharides sample exhibited an intense peak at 3385.45 cm^−1^ attributing to the hydroxyl stretching vibration, a weak peak at 2930.97 cm^−1^ due to C–H stretching vibration and a peak at 1632.23 cm^−1^ which was assigned to the C–O bond of carboxyl group [29]. The absorption peak at 1744.22 cm^−1^ were attributed to the C=O stretching vibration indicating the presence of uronic acids [30], which was in accordance with the fact that PPs had a relatively high uronic acid content. The two characteristic bands at 1100.99 cm^−1^ and 1053.47 cm^−1^ were assigned to the stretching vibrations of (C–OH) side groups and the (C–O–C) glycosidic band vibration, indicating the presence of pyranose in PPs [31]. The stretching peaks at 869.65 and 922.81 cm^−1^ were indicative indexes of α-type glycosidic linkages and d-glucose in pyranose form, respectively [32]. 

**Figure 2:**
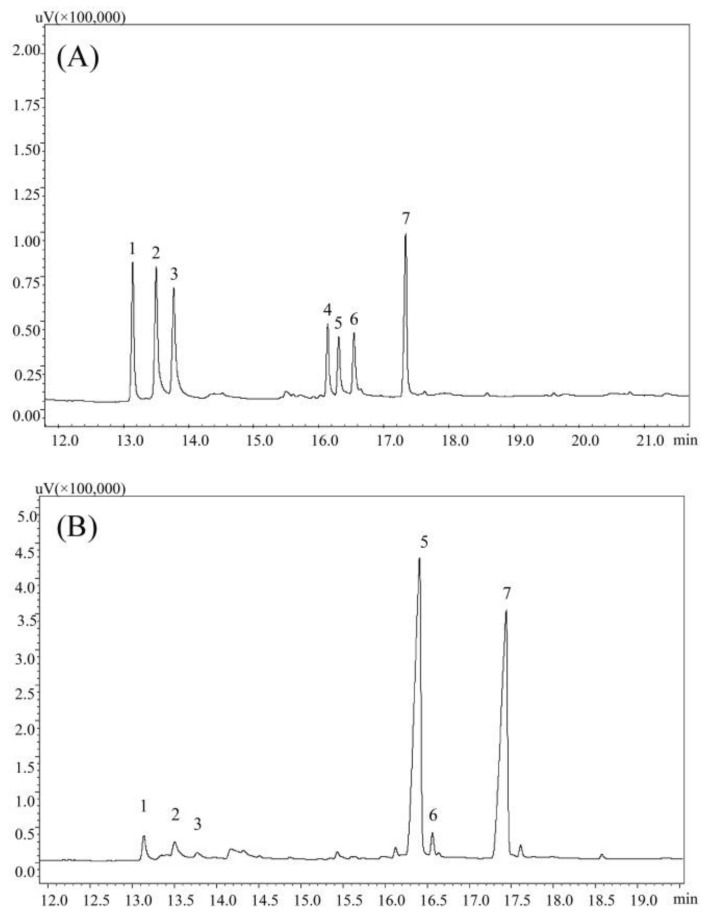
GC chromatograms of alditol acetate derivatives of standard monosaccharide (**A**) and PPs (**B**). Peak identity: 1-rhamnose, 2-arabinose, 3-xylose, 4-mannose, 5-glucose, 6-galactose, 7-internal standard (Inositol).

According to the GC analysis, PPs was composed of rhamnose, arabinose, xylose, glucose and galactose in molar ratio of 0.63:1.00:0.48:11.52:0.57, strongly suggesting that PPs were heterogeneous polysaccharides with the glucose as the major sugar (Figure 2).

### 3.2. In Vivo Antitumor Activity of PPs on S180-Bearing Mice

**Figure 3:**
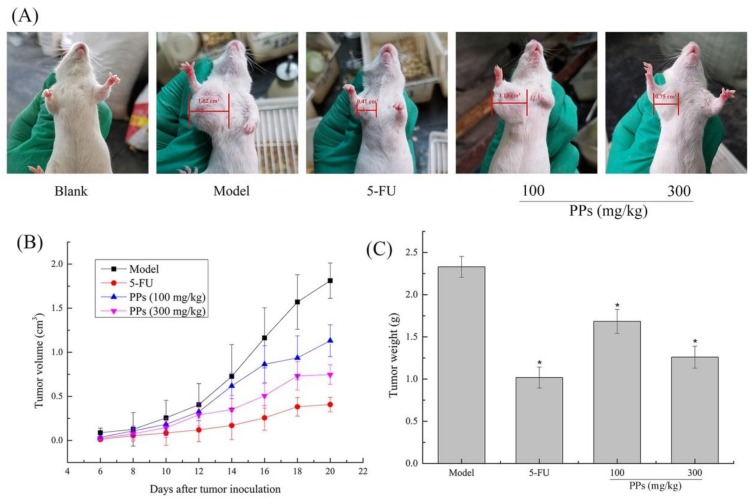
Antitumor effect of PPs on S180-bearing mice. The tumor size of mice after 21 days inoculation of S180 cells (**A**); Effect of PPs on the tumor growth velocity of mice (**B**); Effect of PPs on the tumor weight of mice (**C**). The tumor size in Figure 3A was measured using a vernier caliper. * *p* < 0.05 compared to model group was considered as significant.

The S180 tumor-bearing mice model was established to evaluate the in vivo antitumor activity of PPs (100 and 300 mg/kg). As shown in Figure 3A,B, the tumor size and the tumor growth velocity were higher in model group than those in PPs groups. 5-FU treatment exhibited the lowest tumor size and growth velocity among these different groups. It was also suggested that the antitumor effect of PPs at 300 mg/kg was superior to the dosage of 100 mg/kg, indicating that PPs exhibited prophylactic and inhibitory effect on the formation of S180 solid tumor in a dose-dependent manner. Correspondingly, the tumor weight in model group was 2.33 ± 0.12 g after 21 days inoculation of S180 cells. As compared with model group, the weights of transplanted tumor in PPs groups (100 and 300 mg/kg) were remarkably decreased (*p* < 0.05) with the inhibitory ratio of 27.86% and 50.28%, respectively (Figure 3C). The inhibitory rate of high-dose PPs group was comparable to that of 5-FU group (52.77%). 

The status of thymus and spleen can be used to evaluate the related immune function of the host [33]. As shown in Table 1, there was a significant decrease of thymus index (*p* < 0.05) and an obvious increase of the spleen index (*p* < 0.05) in model group compared to blank group, which could be explained by the fact that the thymus and spleen of model group was seriously damaged under the attack of the tumor [34]. However, significant improvements of thymus and spleen indexes were observed in PPs group as compared to model group (*p* < 0.05), dose-dependently, suggesting that PPs could protect the immune organs of tumor-bearing mice well from S180 tumors. Although, the 5-FU treatment exhibited a relatively high tumor inhibitory rate, the immune organ indexes of the S180-bearing mice in this group was lower than those of the blank group (*p* < 0.05), indicating the toxic effect of 5-FU on immune system in accordance with previous description [7,35].

In terms of the body weight, a significant decrease of the average body weight was observed in the model group compared to the blank group (*p* < 0.05), which might be due to the tumor growth. In contrast, the body weights in the PPs groups, especially in the high-dose group (300 mg/kg) were higher (*p* < 0.05), whereas those in the 5-FU group was relatively lower than those of the model control (*p* < 0.05), showing the side effects of 5-FU on the body weights of tumor-bearing mice. More importantly, the PPs treatment could significantly increase the survival rate of tumor-bearing mice compared to the 5-FU-treated group.

### 3.3. Splenocyte Proliferation and NK Cells Activities Analysis

**Figure 4:**
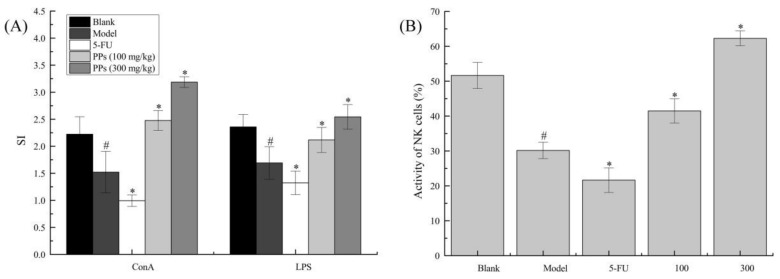
Effect of PPs on ConA- and LPS-induced splenic lymphocyte proliferation (**A**) and the activity of NK cells (**B**) in S180-bearing mice. ^#^ *p <* 0.05 compared to blank group was considered as significant; * *p <* 0.05 compared to model group was considered as significant.

MTT assay was carried out to evaluate the effect of PPs oral administration on splenic lymphocyte proliferation and NK cells activities in S180-bearing mice (Figure 4). As shown, the proliferation of T cells and B cells of model group induced by ConA and LPS, respectively, were significantly decreased compared to blank group (*p* < 0.05). In contrast, the splenocyte proliferation of PPs groups was remarkably increased compared with model group (*p* < 0.05), suggesting that PPs could activate cellular and humoral immune responses to strengthen body function of tumor-bearing mice in a dose-dependent manner. 

As shown in Figure 4B, NK cells activity of model group was significantly reduced compared to blank group (*p* < 0.05). On the contrary, PPs-treatment prominently enhanced NK cells activity as compared with model group (*p* < 0.05). 5-Fu, as an efficacious chemotherapy drug, showed no enhancement on immune system, which was consistent with previous reports [36].

### 3.4. Effects of PPs on Serum Cytokine Levels

ELISA kits were employed to determine the effects of PPs on the expressions of serum IL-2, IFN-γ and TNF-α in S180-bearing mice. As seen in Table 2, the expression of serum IL-2 and IFN-γ in model group was notably reduced while TNF-α was notably increased on account of the transplanted tumor (*p* < 0.05). The levels of IL-2 and TNF-α in serums of the 5-FU-treated mice were appreciably lower than those of the model group (*p* < 0.05), indicated that 5-FU could restrain the expression of cytokines. Moreover, compared to the model group, PPs oral administration significantly increased the expressions of serum IL-2, IFN-γ and TNF-α (*p* < 0.05), dose-dependently. All these data suggested that tumor growth could influence the production and secretion of serum IL-2, TNF-α and IFN-γ, while PPs could enhance the immune function by stimulating the expression of serum cytokines in S180-bearing mice.

### 3.5. Effects of PPs on T Lymphocyte Subsets in Peripheral Blood

The proportions of T cells subsets in peripheral blood from different groups were shown in Table 3. The percentages of CD3^+^ and CD4^+^ cells in model group were observably descended compared to blank group (*p* < 0.05), whereas they were sharply increased (*p* < 0.05) after PPs administration relative to model group, dose-dependently. On the contrary, the mice treated with 5-FU displayed much lower percentages of CD3^+^, CD4^+^ and CD8^+^ T cells when compared with the model group (*p* < 0.05). Accordingly, it was supposed that PPs treatment could restore the immunosuppression induced by tumor cells.

### 3.6. Effects of PPs on Tumor Cells Apoptosis

**Figure 5:**
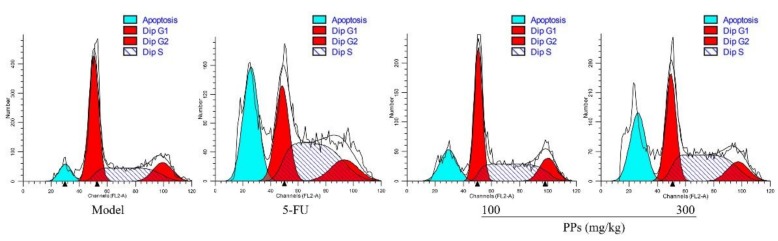
Effects of PPs on cell cycle phase distribution of S180 tumor cells in vivo. The blue, red-1, red-2 colored and shaded areas showed the percentages of cell numbers in apoptosis, G0/G1, G2/M, and S phase, respectively.

To identify whether the tumor growth inhibitory effect of PPs was due to tumor cells apoptosis, the cell cycle arrest of S180 tumor cells were investigated in tumor-bearing mice by flow cytometer. As shown in Figure 5 and Table 4, PPs treatment resulted in a dose-related accumulation of cell numbers in S phase with an increase from 31.15% to 47.35%, and a corresponding decline in the G0/G1 phase. In addition, the percentages of apoptotic DNA in sub-G1 phase of tumor cells were dramatically elevated in the PPs-treated groups (18.08% and 26.21%, respectively, at the dosages of 100 and 300 mg/kg), while the proportions of sub-G1 phase in 5-FU group were up to 31.38%. Our results indicated that PPs could induce S180 tumor cells apoptosis by blocking the cell cycle in S phase.

**Figure 6:**
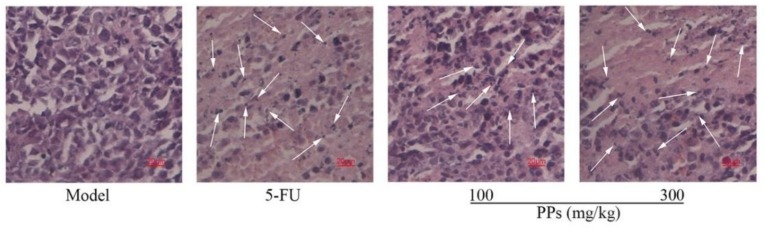
Effects of PPs on the histological morphology of tumor tissues in tumor-bearing mice using H&E staining under optical microscope (magnification, ×400). The arrow heads indicated the apoptotic cell nuclear fragments.

The histological changes of tumor tissues were observed using H&E staining under the optical microscope. Seen from Figure 6, the tumor cells of model control group grew normally and exhibited typical morphological characteristics. In contrast, the tumor tissues of PPs-treated mice showed a lot of apoptotic cells induced by chromatin condensation and nucleus shrinkage. The disruption of tumor cell nucleus and the formation of nuclear fragment could be observed clearly under the treatment with 5-FU or PPs in a dose-dependent manner, indicating the apoptosis of S180 cells. The results of H&E staining further confirmed that PPs could suppress the tumor growth effectively in S180-bearing mice, which was closely related to more S180 tumor cells apoptosis. 

**Figure 7:**
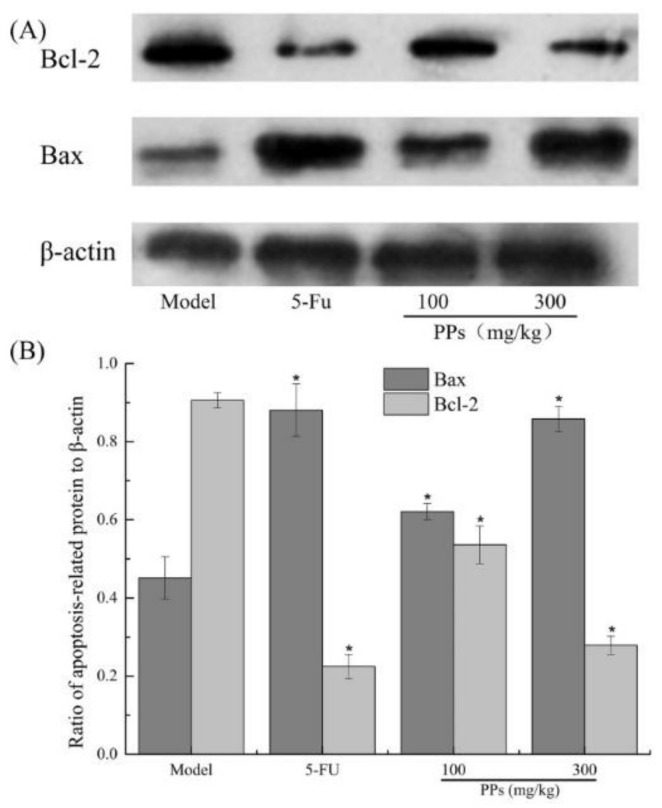
Effect of PPs on Bax and Bcl-2 expression of tumor tissues was observed in tumor-bearing S180 mice by western blotting (**A**); The grayscale ratio of apoptosis-related proteins relative to β-actin protein was analyzed by Quantity One software (**B**). * *p* < 0.05 compared to model group was considered as significant.

As shown in Figure 7, a significant increase of Bax and a remarkable decrease of Bcl-2 expressions were observed in the 5-FU and PPs groups compared to the model group (*p* < 0.05). 

## 4. Discussion

Multiple polysaccharides extracted from various sources have exhibited strong antitumor and immunoregulatory activities on tumor-bearing mice [34,37]. As known, the antitumor and immunoregulatory activities of polysaccharides were related to their monosaccharide composition, molecular weight, uronic acid content and functional groups and so on [38]. In general, polysaccharides with higher molecular mass and good solubility will possess higher bioactivities, and the presence of carboxyl can enhance the antitumor activities of polysaccharides [39]. Moreover, it was reported that α-configuration glycosidic bond in polysaccharides contribute to the antitumor activities [40]. In the present study, PPs were mainly composed of glucose with α-type glycosidic linkages, pyranose rings and uronic acid (16.33%), which significantly inhibited the growth of S180 solid tumors and protect immune organs (thymus and spleen).

As reported, numerous polysaccharides do not have the direct killing effects on cancer cells in vitro [41]. Therefore, the tumor growth inhibition in mice might be activated by the stimulation of the immune response. NK cells belonging to innate immune system have the ability to rapidly respond to malignant cells and participate in immune responses against tumors [42]. Cytokines associated with antitumor immune responses play a critical role in resistance to the tumor growth [5]. T cells become mature and subsequently differentiate into CD4^+^ T cells and CD8^+^ T cells in thymus, and exert antitumor immune response. To the best of our knowledge, CD8^+^ T cells play an important role in recognizing tumor epitopes and eliciting tumor rejection [43], whereas CD4^+^ T cells can provide help for the cytotoxic ability of CD8^+^ T cells on limiting tumor cell growth and the generation of memory CD8^+^ T cells for preventing tumor recurrences [44]. As shown, PPs could remarkably enhance antitumor immunity via improving the killing activity of NK cells, recognition capability of CD4^+^ T cells and cytotoxic ability of CD8^+^ T cells, and increase the expression levels of IL-2, IFN-γ and TNF-α in serum, finally leading to the inhibitory effect on S180 tumors.

In order to demonstrate the antitumor mechanisms of PPs on S180 tumor-bearing mice, the tumors in different groups were taken from mice and further investigated. The Bcl-2 family proteins comprise anti-apoptotic (Bcl-2) and pro-apoptotic gene (Bax), which play a vital role in the progression of apoptosis, especially via intrinsic or mitochondrial pathway [45]. The balance between the Bax and Bcl-2 proteins is crucial to the release of apoptosis inducing factors from the mitochondria, and an imbalance of these two signaling proteins can result in the disruption of mitochondrial outer membrane, which further leads to cell apoptosis. Take these experiments about cell apoptosis of S180 solid tumor cells into consideration, we confirmed that PPs could induce the apoptosis of S180 cells via arresting the cell cycle in S phase, down-regulating the Bcl-2 expressions and up-regulating the Bax levels. As we all know, 5-FU can activate thymidylate synthase-inhibiting fluorodeoxyuridine, thereby prevent DNA synthesis and further lead to cell apoptosis [46]. In the present study, 5-FU directly inhibited S180 cells proliferation with obviously immunosuppressive effects on tumor-bearing mice. Therefore, PPs showed preferable practical application in food and medical industries.

## 5. Conclusions

In this study, a water-soluble acidic heteropolysaccharides (PPs) was obtained from pomelo peels, which was mainly composed of glucose and had α-type glycosidic linkages and pyranose form. The PPs was confirmed to be consisted of a high content of total sugar (74.52%) and uronic acid (16.33%) with no significant proteinous materials. The in vivo antitumor experiments showed that PPs could effectively inhibit the malignant proliferation of transplanted S180 tumor in mice with little toxicity to the host. The potential antitumor mechanism of PPs was further investigated, and the result shown that PPs could effectively improve the spleen and thymus indexes, enhance the spleen lymphocyte proliferation and NK cells activities, as well as increase the levels of serum cytokine and the proportion of CD4^+^ T cells in peripheral blood. PPs were also shown to induce the transplanted S180 tumor cells apoptosis by arresting the cell cycle in S phase, down-regulating the Bcl-2 expressions and up-regulating Bax levels. All these results suggested that the in vivo antitumor effects treated with PPs might be attributed to the polysaccharides’ immunoregulatory properties and apoptosis-inducing characters on the tumor-bearing mice. These fundamental researches about the physiochemical properties and their related antitumor mechanisms of pomelo peels polysaccharides may be helpful to the development of PPs as supplement or antitumor agents.

## Figures and Tables

**Table 1:** Effects of PPs on the body weight, immune organs indexes of S180-bearing mice.

Treatment	Dose (mg/kg)	Increased of Body Weight (g)	Thymus Index (mg/g)	Spleen Index (mg/g)	Numbers Start/End
Blank	-	4.25 ± 1.28	2.64 ± 0.18	4.60 ± 0.29	10/10
Model	-	3.26 ± 0.91 ^#^	1.67 ± 0.35 ^#^	7.15 ± 0.25 ^#^	10/9
5-Fu	20	1.25 ± 0.99 *	1.27 ± 0.17 *	3.87 ± 0.19 *	10/8
PPs	100	3.74 ± 0.80	2.09 ± 0.28 *	5.83 ± 0.22 *	10/10
	300	4.45 ± 1.39 *	2.53 ± 0.28 *	4.58 ± 0.16 *	10/10

^#^ *p* < 0.05 compared with the blank group; * *p* < 0.05 compared with the model group.

**Table 2:** Effect of PPs on the levels of IL-2, IFN-γ and TNF-α in serum of S180-bearing mice.

Treatment	Dose (mg/kg)	IL-2 (pg/mL)	IFN-γ (pg/mL)	TNF-α (pg/mL)
Blank	-	4.76 ± 0.13	20.54 ± 2.56	11.93 ± 2.13
Model	-	2.71 ± 0.16 ^#^	8.98 ± 3.21 ^#^	17.14 ± 2.24 ^#^
5-Fu	20	1.28 ± 0.12 *	10.68 ± 1.83	10.21 ± 1.94 *
PPs	100	3.90 ± 0.15 *	20.04 ± 2.31 *	22.13 ± 2.82 *
	300	5.12 ± 0.25 *	31.34 ± 4.09 *	27.90 ± 2.92 *

^#^ *p* < 0.05 compared with the blank group; * *p* < 0.05 compared with the model group.

**Table 3:** Effects of PPs on the percentages of T lymphocyte subsets in peripheral blood of tumor-bearing mice.

Treatment	Dose (mg/kg)	CD3^+^ (%)	CD4^+^ (%)	CD8^+^ (%)
Blank group	-	64.38 ± 5.13	43.43 ± 4.15	23.89 ± 3.33
Model group	-	46.12 ± 4.32 ^#^	27.68 ± 2.02 ^#^	21.60 ± 2.73
5-Fu	20	40.11 ± 1.87 *	21.51 ± 1.87 *	15.60 ± 1.21 *
PPs	100	50.09 ± 3.24 *	32.35 ± 2.54 *	21.98 ± 2.23
	300	60.75 ± 5.18 *	43.59 ± 3.76 *	22.24 ± 3.11

^#^ *p* < 0.05 compared with the blank group; * *p* < 0.05 compared with the model group.

**Table 4:** Effects of PPs on cell cycle phase distribution of S180 tumor cells in vivo.

Treatment	Dose (mg/kg)	G0/G1 (%)	S (%)	G2/M (%)	Apoptosis (%)
Model	-	53.19 ± 1.57	31.15 ± 1.85	15.66 ± 1.02	8.54 ± 1.23
5-Fu	20	34.53 ± 2.12 *	50.06 ± 2.32 *	15.41 ± 0.87	31.38 ± 2.16 *
PPs	100	46.01 ± 1.66 *	38.23 ± 3.22 *	15.76 ± 1.91	18.08 ± 1.24 *
	300	38.71 ± 2.77 *	47.35 ± 2.73 *	13.94 ± 0.94 *	26.21 ± 1.17 *

* *p* < 0.05 compared with the model group.

## Abbreviations

PPs	Pomelo peels polysaccharides
MWCO	Molecular weight cut-off
FT-IR	Fourier transform infrared spectroscopy
GC	Gas chromatography
TFA	Trifluoroacetic acid
5-FU	5-fluorouraci
FBS	Fetal bovine serum
ConA	Concanavalin A
LPS	Lipopolysaccharide
NK	Natural killer
IL-2	Interleukin-2
IFN-γ	Interferon-γ
TNF-α	Tumor necrosis factor-α
BRM	Biological response modifier
MTT	3-(4,5-dimethylthiazol-2-yl)-2,5diphenyltetrazolium bromide
DMSO	Dimethyl sulfoxide
FBS	Fetal bovine serum
H&E	Hematoxylin and eosin
PI	Propidium iodide
FCM	Flow cytometry
RIPA	Radio immunoprecipitation assay
BCA	Bicinchoninic acid
ECL	Enhanced chemiluminescence
WB	Western blotting
